# Mechanical Differences between Men and Women during Overground Load Carriage at Self-Selected Walking Speeds

**DOI:** 10.3390/ijerph19073927

**Published:** 2022-03-25

**Authors:** Kane Middleton, Danielle Vickery-Howe, Ben Dascombe, Anthea Clarke, Jon Wheat, Jodie McClelland, Jace Drain

**Affiliations:** 1 Discipline of Sport and Exercise Science, School of Allied Health, Human Services and Sport, La Trobe University, Bundoora 3086, Australia; d.vickery-howe@latrobe.edu.au (D.V.-H.); a.clarke@latrobe.edu.au (A.C.); 2 Applied Sport Science and Exercise Testing Laboratory, School of Life and Environmental Sciences, University of Newcastle, Ourimbah 2258, Australia; ben.dascombe@newcastle.edu.au; 3 Academy of Sport and Physical Activity, Sheffield Hallam University, Sheffield S10 2BP, UK; j.wheat@shu.ac.uk; 4 Discipline of Physiotherapy, School of Allied Health, Human Services and Sport, La Trobe University, Bundoora 3086, Australia; j.mcclelland@latrobe.edu.au; 5 Land Division, Defence Science and Technology Group, Fishermans Bend 3207, Australia; jace.drain@dst.defence.gov.au

**Keywords:** walking gait, spatiotemporal, kinematics, kinetics, military

## Abstract

Few studies have directly compared physical responses to relative loading strategies between men and women during overground walking. This study aimed to compare gait mechanics of men and women during overground load carriage. A total of 30 participants (15 male, 15 female) completed three 10-min walking trials while carrying external loads of 0%, 20% and 40% of body mass at a self-selected walking speed. Lower-body motion and ground reaction forces were collected using a three-dimensional motion capture system and force plates, respectively. Female participants walked with a higher cadence (*p* = 0.002) and spent less absolute time in stance (*p* = 0.010) but had similar self-selected walking speed (*p* = 0.750), which was likely due to the female participants being shorter than the male participants. Except for ankle plantarflexion moments, there were no sex differences in spatiotemporal, kinematic, or kinetic variables (*p* > 0.05). Increasing loads resulted in significantly lower self-selected walking speed, greater stance time, and changes in all joint kinematics and kinetics across the gait cycle (*p* < 0.05). In conclusion, there were few differences between sexes in walking mechanics during overground load carriage. The changes identified in this study may inform training programs to increase load carriage performance.

## 1. Introduction

Load carriage is a common activity within the military and the success of a mission can be affected by the soldier’s ability to undertake this task, noting that they are likely required to perform other tasks whilst bearing load [1,2]. Acknowledging the potential deleterious effect of load carriage on military task performance during World War I, there was a subsequent recommendation to limit load to 33% of body mass (BM) [3,4]. However, setting a load limit for soldiers based on body mass is not practical, as operational requirements (e.g., mission duration, threat level, role) will dictate the load-carriage requirements. Recent operational data demonstrates that soldiers carry loads considerably heavier than this proposed load limit [1,2].

Load carriage has been repeatedly associated with musculoskeletal injury risk [5,6,7,8]. Recent evidence from deployed U.S. Army soldiers showed that ~10% of all musculoskeletal injuries were sustained during dismounted patrolling [9]. This is consistent with evidence from Australian Army soldiers, which demonstrated that 8% of reported injuries were sustained during load carriage [10]. The literature also consistently demonstrates that women have a higher risk of musculoskeletal injury compared with men during both training and operations [9,11,12,13]. However, when aerobic fitness is accounted for, these differences in injury rates between women and men reduce considerably [14,15], except for stress fractures [16,17]. Given sex restrictions in direct combat roles are progressively being removed [6,18], women are increasingly being exposed to injury and health risks from heavy load carriage. An increased understanding of the biomechanical responses to load carriage between sexes may facilitate improved task management, reduce injury incidence [14,15,19,20] and decrease the number of lost working days.

Although female recruits seem to be at greater risk of injury during military training, there is limited research comparing the biomechanical responses of men and women during load carriage [21,22]. When assessing both sexes carrying 0%, 10%, 20% and 30% of body mass on a treadmill, Silder et al. [22] did not report any sex differences in any spatiotemporal, kinematic, kinetic or muscle activation data. Krupenevich et al. [21] suggested that these similarities between sexes was due to the relative rather than absolute loads carried, and consequently the researchers investigated sex differences using a standardised load of 22 kg while walking overground. Their hypotheses were generally not supported with the only difference being a 2° greater trunk forward lean in female participants when compared with male participants. The authors suggested that the standardised load may have been too light to elicit sex differences. The average 22 kg load carried in Krupenevich et al. [21] was similar or slightly larger than the average loads used by Silder et al. [22] at 30% of body mass (male participant’s average BM was 75 kg, with 30% being 22.5 kg; female participant’s average body mass was 63 kg with 30% being 18.9 kg). In the field, it is likely that individuals may need to carry loads greater than this, therefore there is a need to use greater loads during overground walking conditions to confirm whether sex differences are present.

There is little research comparing the physical responses of men and women to load carriage. The aims of the current study were to determine whether there are differences in gait mechanics between male and female participants during overground load carriage across a range of relative loads up to 40% of body mass. It was hypothesised that male and female participants with no load carriage experience would demonstrate similar spatiotemporal measures, kinematics and kinetics when carrying external loads of up to 40% body mass while walking overground at a self-selected speed.

## 2. Materials and Methods

### 2.1. Participants

Fifteen self-reported female (age: 25.1 ± 6.1 y, height: 1.65 ± 0.07 m, body mass: 61.5 ± 6.9 kg) and 15 male (age: 22.3 ± 2.3 y, height: 1.79 ± 0.07 m, body mass: 74.2 ± 8.5 kg) participants were recruited into this study. All participants reported no known gait abnormalities, were injury free for at least six months prior to participating, and had no previous occupational load carriage experience. All participants provided written informed consent to ethical procedures approved by La Trobe University’s Science, Health and Engineering College Human Ethics Sub-Committee (Ethics protocol#: HEC18146). This same sample participated in related studies published previously [23,24].

### 2.2. Experimental Protocol

This study was a secondary analysis of data reported previously [23]. Participants completed ten-minute overground walking trials on a ‘figure 8’ track that allowed a 15 m straight section through the capture volume. Trials were completed at each of three relative body-borne loads (0%, 20%, and 40% BM) with at least 10-min passive rest between trials. For each walking trial, the control condition (0% BM) was performed first, with load incremented (20% BM followed by 40% BM) to ensure safe task completion. The load was added in the form of a weighted vest, with equal distribution between front and back to keep the load close to the centre of mass; this being most comparable to the double pack [25]. Average walking speed was calculated from lap times and had to be within 5% of the previously identified self-selected walking speed to be considered a valid trial. Participants were provided verbal feedback if they were required to modify their walking speed.

### 2.3. Motion Capture

A total of 36 retroreflective markers were attached to each participant’s pelvis and lower limbs. Markers were attached bilaterally on the following anatomical landmarks: anterior and posterior superior iliac spines, left and right iliac crests, medial and lateral femoral epicondyles, medial and lateral malleoli, calcaneus, first metatarsal head, and the fifth metatarsal head. Four additional markers were affixed to custom molded thermoplastic plates and attached laterally on each thigh and lower leg to measure segment motion during walking trials. Marker trajectories were captured with a 10-camera Vicon T-16 opto-reflective motion capture system (Vicon Motion Systems Ltd., Oxford, UK; 100 Hz) while ground reaction forces (GRF) were captured with a 400 × 600 mm ground-embedded force platform (BP400600-OP; Advanced Mechanical Technology, Inc., Watertown, MA, USA; 1000 Hz). Raw trajectory and force data were filtered using a dual-pass second order low-pass Butterworth filter, with the cutoff frequency (f_c_ = 6 Hz) determined by a residual analysis and visual inspection [26,27,28].

**Figure A1:**
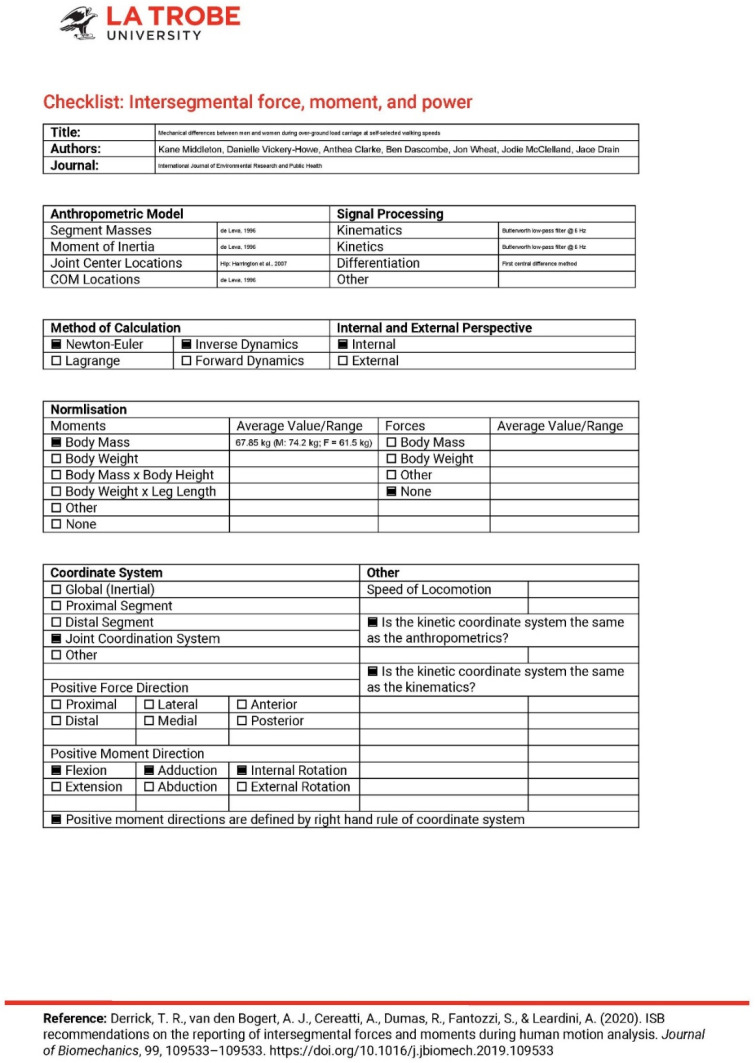
Checklist for the reporting of intersegmental moments.

A seven-segment lower limb and pelvis direct kinematic model was used to calculate required joint centres [23]. Hip joint centres were calculated using the regression equation of Harrington et al. [29], while the knee and ankle joint centres were determined by taking the midpoint between the femoral epicondyles and malleoli, respectively. Segment-embedded anatomical coordinate systems were defined following the International Society of Biomechanics recommendations, while non-orthogonal joint coordinate systems were used to calculate sagittal plane hip, knee and ankle flexion-extension joint angles [30]. Segment kinematics and inertial properties along with GRFs were used to perform inverse dynamic analyses within the model to calculate internal joint moments and were reported in the respective non-orthogonal joint coordinate system [31,32]. All joint moments and GRFs were normalised to BM. Joint kinematics and kinetics were also temporally normalised to the length of the gait cycle (heel strike to heel strike; [33]). Refer to Appendix A (Figure A1) for more details [34].

### 2.4. Statistical Analyses

For discrete spatiotemporal variables, the mean of three strides from each participant were calculated for analyses. Data were screened for normality and sphericity prior to any analysis being conducted. Independent samples *t* tests were performed to investigate differences in demographic data between sexes. Cohen’s *d* values are reported as the *t* test effect size.

Mixed-design ANOVAs were performed to investigate the interaction and main effects of sex (female, male) and load magnitude (0%, 20%, 40% BM). Partial eta-squared (η^2^_p_) values are reported as the ANOVA effect size. An alpha value of 0.05 was used to determine when post hoc independent or paired-sample *t* tests were to be performed.

Four-stage hierarchical multiple linear regression analyses were performed to determine the influence of physical characteristics (sex, height, mass, and age) on the spatiotemporal outcome variables that were found to be significantly different between sexes in the ANOVA (*p* < 0.05). Entry order was based on the strength of bivariate correlations between each independent variable and the dependent variable. Cohen’s *f*^2^ values are reported as the regression effect size. All discrete data were analysed using jamovi (version 2.0.0.0, The jamovi project [35]).

For continuous data, statistical parametric mapping (SPM) mixed-design analyses of variance were performed to investigate the interaction and main effects of sex (female, male) and load magnitude (0%, 20%, 40% BM) across the gait cycle. An alpha value of 0.05 was used for interactions and main effects to determine when *post hoc* independent or paired-sample *t* tests with Bonferroni corrections were to be performed. All continuous data were analysed using the spm1D package (v. 0.4.8, https://spm1d.org/, [36]) (accessed on 18 October 2021)in MATLAB (R2021a, The MathWorks Inc., Natick, MA, USA).

Data are presented as mean ± standard deviation (M ± SD) unless otherwise stated.

## 3. Results

Female participants were older, shorter, lighter, and carried less absolute external load than the male participants (Table 1). The average absolute mass of the 20% and 40% BM conditions were representative of Australian Army patrol and marching order (light) operational loads, respectively.

Self-selected walking speed was similar between sexes and decreased by 0.15 km/h (~3%) in the 40% BM condition compared with the other conditions (Table 2). Cadence was 9 ± 3 steps/min (8%) higher in the female participants compared with the male participants and decreased by 2 steps/min (~2%) for the female participants in the 40% BM condition compared with the other conditions. Stride length and width was consistent across conditions. Absolute stance time was 0.05 s (8%) lower in the female participants compared with the male participants but was similar as a temporal proportion of the gait cycle. Both absolute and relative stance time increased with increasing load.

Cadence was correlated with height (*r* = −0.543, *p* = 0.002), body mass (*r* = −0.446, *p* = 0.013), and age (*r* = 0.435, *p* = 0.016). A hierarchical multiple regression with cadence as the dependent variable (Table 3) revealed that in stage one, height accounted for 29.5% of the variation (F (1,28) = 11.73, *f*^2^ = 0.418, *p* = 0.002). Subsequent stages revealed that body mass explained an additional 2.0% of variation (F (1,27) = 0.77, *f*^2^ = 0.029, *p* = 0.387) and age an additional 19.6% (F (1,26) = 10.42, *f*^2^ = 0.401, *p* = 0.003). Sex did not explain any additional variance in cadence (F (1,25) = 0.01, *f*^2^ = 0.000, *p* = 0.938).

Stance time was correlated with height (*r* = 0.492, *p* = 0.006) and body mass (*r* = 0.477, *p* = 0.008) but not age (*r* = −0.282, *p* = 0.132). A hierarchical multiple regression with stance time as the dependent variable (Table 4) revealed that in stage one, height accounted for 24.2% of the variation (F (1,28) = 8.94, *f*^2^ = 0.319, *p* = 0.006). Subsequent stages revealed that body mass explained an additional 4.8% of variance (F (1,27) = 1.84, *f*^2^ = 0.068, *p* = 0.186), age an additional 7.8% (F (1,26) = 3.19, *f*^2^ = 0.123, *p* = 0.086), and sex an additional 0.1% (F (1,25) = 0.06, *f*^2^ = 0.002, *p* = 0.801).

**Figure 1:**
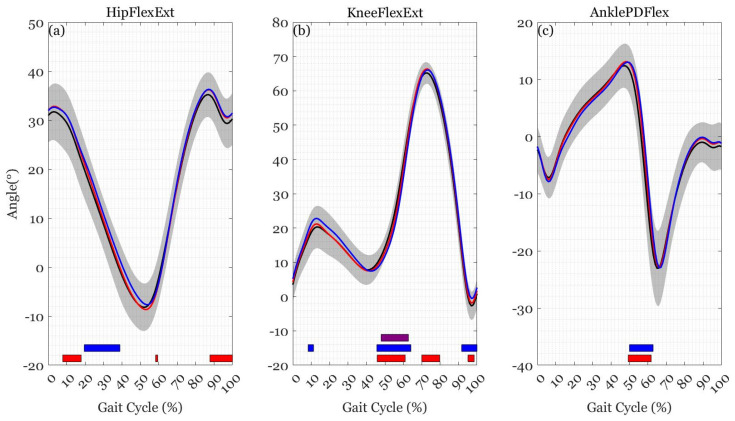
Mean trajectories for (**a**) hip joint flexion-extension angle, (**b**) knee joint flexion-extension angle, and (**c**) ankle joint plantar-dorsiflexion angle during load carriage of 0% BM (black), 20% BM (red), and 40% BM (blue). The colored bars show when the SPM {*t*} critical threshold was exceeded between 0% BM and 20% BM (red), 0% BM and 40% BM (blue), and 20% BM and 40% BM (purple).

No sex-by-load interactions nor main effects for sex were found for any kinematic variable (*p* > 0.05). There was a main effect of load at the hip during mid-to-late stance (15–47%, F(2,56) > 5.420, *p* = 0.002), at the knee during late stance (44–65%) as well as from late swing through to early stance (88–15%, F(2,56) > 6.200, *p* < 0.008), and at the ankle during late stance and early swing (49–64%) as well during late swing (85–93%, (F(2,56) > 5.945, *p* < 0.031). Paired-sample comparisons revealed differences between loads across the gait cycle (Figure 1 and Table A1 [Appendix A]).

**Figure 2:**
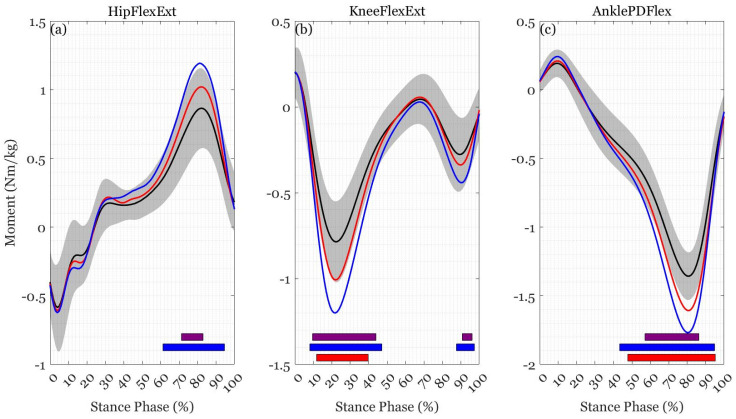
Mean trajectories for (**a**) hip joint flexion-extension moment, (**b**) knee joint flexion-extension moment, and (**c**) ankle joint plantar-dorsiflexion moment during load carriage of 0% BM (black), 20% BM (red), and 40% BM (blue). The colored bars show when the SPM {*t*} critical threshold was exceeded between 0% BM and 20% BM (red), 0% BM and 40% BM (blue), and 20% BM and 40% BM (purple).

**Figure 3:**
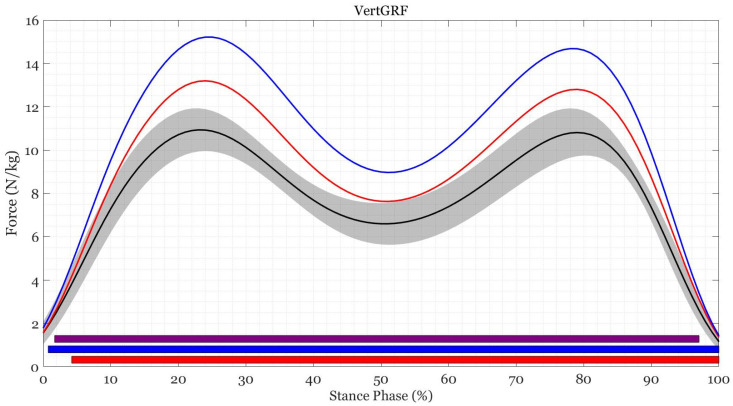
Mean trajectories for vertical ground reaction force during load carriage of 0% BM (black), 20% BM (red), and 40% BM (blue). The colored bars show when the SPM {*t*} critical threshold was exceeded between 0% BM and 20% BM (red), 0% BM and 40% BM (blue), and 20% BM and 40% BM (purple).

No sex-by-load interactions were found for any kinetic variable (*p* > 0.05). There was a main effect of load at the hip during mid-to-late stance (57–95%, F(2,52) > 6.403, *p* < 0.001), at the knee during early-to-mid stance (7–50%) and late stance 85–98% (F(2,52) > 6.023, *p* < 0.014), at the ankle during mid-to-late stance (42–96%, F(2,52) > 5.937, *p* < 0.001), and for vertical GRF during the entire stance phase (0–100%, F(2,56) > 6.124, *p* < 0.001). *Post hoc* comparisons revealed differences between loads throughout the gait cycle (Figure 2 and Figure 3, Table A2 [Appendix A]). There was a main effect of sex at the ankle during mid stance (41–62%, F(1,26) > 9.493, *p* < 0.001), with male participants having larger plantarflexion moments than female participants during mid stance (32–68%, *t* > 2.872, *p* < 0.001).

## 4. Discussion

There were no sex-by-load interactions for any spatiotemporal, kinematic, or kinetic variable during overground load carriage at self-selected walking speeds. Despite a smaller height, female participants had a similar self-selected walking speed to male participants, which was a result of a higher cadence and spending less time in stance phase. Through the multiple linear regression analyses, it was shown that these differences were predominantly explained by participant height, not sex. There were no sex differences for any kinematic variable across the gait cycle, whereas male participants had higher ankle plantar flexion moments than female participants during mid stance. Increasing loads resulted in lower self-selected walking speed, greater stance time, as well as changes in all joint kinematics and kinetics across the gait cycle.

The significantly higher cadence and lower time in stance in the female participants can be partially explained by the relationship between walking speed (similar), height (with the female participants being shorter), and leg length. The Australian Warfighter Anthropometry Survey found that height (Female: 1.65 m, Male: 1.78 m) and leg length (Female: 0.93 m, Male: 0.99 m) were similarly proportional between male and female participants [37]. Given the similar relative stride length between the two cohorts, the female participants, or more generally shorter people irrespective of sex, would need to have a higher cadence for a given walking speed. This increased cadence would result in a greater number of ground impacts per foot [38,39]. Although from a mechanobiological perspective the magnitude of strain is weighted more heavily than the number of loading cycles [40,41], these additional foot strikes and less time to attenuate the GRF may lead to higher cumulative bone load [42]. This could be particularly detrimental in the field when absolute rather than relative loads are carried, and could be a potential mechanism for the higher incidence of stress fractures in female soldiers [43,44,45]. The generally shorter heights of women has been suggested to increase their risk of stress fractures [46] however previous research in women is equivocal [47,48] and a positive association between height and stress fracture risk has been reported in men [49]. As advocated by Gill et al. [50], further research is required that discerns sex and physical characteristics (e.g., anthropometric, strength, musculoskeletal loading) on biomechanical adaptation to load carriage and determinants of injury risk, and subsequent mitigation strategies, in military training.

The addition of load decreased self-selected walking speed and increased both relative and absolute stance time. This agrees with previous literature whereby increased stance time is an adaptation to increase gait stability while carrying load and maintaining forward progression [39,51,52,53,54]. Previous research has shown decreases in step length and increases in step rate with the addition of external load primarily in a backpack [53,54,55]. The current study did not find any change in these measures as external load increased, suggesting that participants largely maintained the spatiotemporal structure of their gait pattern across the trial conditions. However, the vest loading strategy employed in the current study (distributed evenly between front and back) may have allowed the participants to maintain spatiotemporal variables more similarly to unloaded walking when compared to a backpack [56].

Previous research has shown no kinematic differences between sexes during load carriage [21,22]. The results of the current study support these previous findings and demonstrate that despite the female participants being shorter (14 cm) and lighter (12.7 kg) than the male participants, they responded similarly to the addition of relative external load. The absence of sex differences has been suggested to be a contributing factor to increased injury risk, whereby women do not adapt walking mechanics when carrying load to account for their shorter height, lower body mass, and lower muscle strength [21]. During an absolute loading strategy of 22 kg, women have exhibited a small (2°) increase in trunk lean; however, when exploring the data by body mass it was found that lighter individuals had more trunk lean and less propulsive forces when compared with the heavier group of participants [21]. These results further support the notion that anthropometrics and strength may influence load carriage performance more than sex.

The increased hip and knee flexion during late swing in the 20% BM and 40% BM conditions suggests that the participants used a preparatory strategy to accommodate an increased mass during early stance. This was reflected by the subsequent increases in joint flexion angles of the hip and knee during early-to-mid stance, supporting previous research [22,57,58]. These kinematic changes could be attributed to an attempt at lowering the body’s centre of mass, allowing more time for skeletal muscle to dissipate impact forces and decrease injury risk [52,56,59,60]. This is also reflected by the increase in absolute and relative stance time that additionally increases stability and time in double support while walking with load [61]. The lower knee flexion during late stance is likely to be a consequence of the increased knee extension range that would aid in forward propulsion. Overall, kinematic changes due to increased external load seem to occur to control the body’s centre of mass and attenuate GRFs during stance.

Vertical GRF increased in proportion to the addition of external load and was the outcome of this external load rather than change in spatiotemporal parameters [22,56,62,63]. Greater ankle plantarflexion, knee extension, and hip flexion moments are required to counteract additional stress on musculature around the joints whilst carrying loads like those in the current study [22,52,57]. It appears that the addition of external load requires knee extension moments to increase to counteract the increased load and subsequent vertical GRF during weight acceptance. This is followed by an increase in ankle plantar flexion moment and hip flexion moment during mid-to-late stance, and increased knee extension moment during late stance to maintain and produce forward progression and propulsion. The larger plantarflexion moment demonstrated by the male participants was likely a consequence of greater absolute loads and the moment normalisation to body mass, which did not include the external load or account for the differences in height between male and female participants. While greater demand on the musculature to control the knee and ankle may be a potential source for injury at these sites [64], increases in hip flexion, knee extension and ankle plantarflexion strength may be protective and improve load carriage performance.

The load distribution used in this study was delimited to being an equal anterior–posterior distribution to eliminate distribution as a potential confounding factor and was representative of wearing armour, pouches, or a double pack. Therefore, these results are most relevant for military application or similar load carriage scenarios. Backpack carriage may cause different gait adaptations to those in this study when considering posterior load carriage [61]. Further research is required in recruit populations with no prior load carriage experience to monitor biomechanical adaptations during short-term absolute load carriage, prolonged load carriage, and through initial recruit and employment training to gauge how military personnel respond to load with increasing experience. Although the removal of confounding factors such as body mass and physical size by carrying relative loads and walking at self-selected walking speeds enabled the current investigation of sex differences during load carriage, this does not reflect practice within a military setting. *Bona fide* occupational requirements result in absolute loads and fixed walking speeds being adopted, irrespective of age or sex [65]. It is likely that when comparing greater absolute loads and walking speeds, sex differences may manifest [21]. However, even when using absolute loading strategies, sex differences may be a result of anthropometric differences such as height and mass. Future research that investigates walking mechanics during prolonged load carriage tasks would be more representative in understanding if sex, or size, differences become amplified with fatigue and for durations greater than 10 min. An increased understanding of the biomechanical responses to load carriage between sexes, and more importantly, the influence of physical characteristics (e.g., height, body mass, lean mass), may facilitate improved load carriage conditioning and task management, and/or help to mitigate injury in at-risk cohorts [14,15,19,20]. These outcomes will ultimately help to reduce the number of working days lost to injury or disability in both recruits and active-duty soldiers.

## 5. Conclusions

Female participants displayed greater cadence and decreased stance time when compared with male participants during ten minutes of overground load carriage with external loads up to 40% BM. However, these differences were likely the result of differences in anthropometry, rather than sex. All other spatiotemporal, kinematic, and kinetic variables were similar between sexes. With the addition of load, an increase in stance time and changes to joint angles and kinetics were evident to counteract and control the additional external load.

## Figures and Tables

**Table 1:** Demographic and load differences between the female and male participants. BM: body mass.

	Female	Male	Mean Difference [95% CI]	Effect Size (Cohen’s *d*) [95% CI]	*t* Statistic	*p* Value
	(Mean ± SD)					
Age (years)	25.1 ± 6.1	22.3 ± 2.3	−2.8 [−6.3, 0.6]	−0.619 [−1.36, 0.142]	−1.7	0.101
Body mass (kg)	61.5 ± 6.9	74.2 ± 8.5	12.7 [7.0, 18.5]	1.653 [0.698, 2.574]	4.53	< 0.001
Height (cm)	166 ± 7	179 ± 7	13 [8, 19]	1.878 [0.867, 2.853]	5.14	<0 .001
External Load (20% BM)	12.2 ± 1.3	14.9 ± 1.8	2.7 [1.5, 3.9]	1.661 [0.704, 2.584]	4.55	< 0.001
External Load (40% BM)	24.5 ± 2.7	29.9 ± 3.6	5.4 [3.0, 7.8]	1.706 [0.738, 2.639]	4.67	<0.001

**Table 2:** Spatiotemporal measures in female and male participants across overground walking trials. Data presented as mean ± SD. BM: body mass.

	0% BM		20% BM		40% BM		Effect Size *(η^2^_p_)*			*p* Value		
	Female	Male	Female	Male	Female	Male	Sex * Load	Sex	Load	Sex * Load	Sex	Load
Walking speed (km/h)	4.8 ± 0.5	4.7 ± 0.5	4.8 ± 0.5	4.7 ± 0.5	4.6 ± 0.5	4.6 ± 0.5	0.058	0.004	0.234	0.193	0.750	0.001
Cadence (steps/min)	118 ± 5	109 ± 7	118 ± 7	108 ± 7	116 ± 7	109 ± 8	0.086	0.294	0.043	0.098	0.002	0.286
Stride length (% height)	82 ± 9	83 ± 6	82 ± 8	82 ± 7	81 ± 11	78 ± 12	0.021	0.002	0.054	0.513	0.830	0.219
Step width (% height)	7 ± 1	6 ± 1	6 ± 1	6 ± 2	7 ± 1	6 ± 2	0.014	0.056	0.019	0.681	0.207	0.584
Stance time (s)	0.61 ± 0.04	0.66 ± 0.05	0.62 ± 0.04	0.68 ± 0.06	0.64 ± 0.05	0.69 ± 0.07	0.084	0.225	0.423	0.106	0.008	<0.001
Stance time (%)	60 ± 2	60 ± 2	61 ± 2	61 ± 2	62 ± 2	62 ± 1	0.044	0.000	0.718	0.281	0.914	<0.001

**Table 3:** Regression results for cadence.

Model	Estimate (B)	SE (B)	*β*	*t*	*p*	*R*	*R* ^2^	Δ*R*^2^
Stage 1						0.543	0.295	-
Intercept	190.423	22.650	-	8.41	<0.001			
Height	−0.449	0.131	−0.543	−3.43	0.002			
Stage 2						0.561	0.315	0.020
Intercept	184.510	23.714	-	7.78	<0.001			
Height	−0.358	0.167	−0.433	−2.14	0.042			
Mass	−0.144	0.164	−0.178	−0.88	0.387			
Stage 3						0.715	0.511	0.196
Intercept	170.732	20.859	-	8.18	<0.001			
Height	−0.399	0.145	−0.483	−2.76	0.011			
Mass	−0.099	0.142	−0.123	−0.70	0.491			
Age	0.751	0.233	0.445	3.23	.003			
Stage 4						0.715	0.511	0.000
Intercept	172.349	29.544	-	5.83	<0.001			
Height	−0.407	0.179	−0.492	−2.28	0.032			
Mass	−0.104	0.157	−0.128	−0.66	0.514			
Age	0.761	0.267	0.451	2.85	0.009			
Sex (F-M)	−0.298	3.778	−0.037	−0.08	0.938			

**Table 4:** Regression results for stance time.

Model	Estimate (B)	SE (B)	*β*	*t*	*p*	*R*	*R* ^2^	Δ*R*^2^
Stage 1						0.492	0.242	-
Intercept	0.161	0.164	-	0.99	0.332			
Height	0.003	0.001	0.492	2.99	0.006			
Stage 2						0.539	0.290	0.048
Intercept	0.226	0.168	-	1.34	0.190			
Height	0.002	0.001	0.319	1.55	0.134			
Mass	0.002	0.001	0.280	1.36	0.186			
Stage 3						0.606	0.368	0.078
Intercept	0.286	0.165	-	1.73	0.095			
Height	0.002	0.001	0.350	1.76	0.091			
Mass	0.001	0.001	0.245	1.23	0.231			
Age	−0.003	0.002	−0.280	−1.79	0.086			
Stage 4						0.608	0.369	0.001
Intercept	0.245	0.234	-	1.05	0.304			
Height	0.002	0.001	0.385	1.57	0.129			
Mass	0.001	0.001	0.266	1.21	0.238			
Age	−0.004	0.002	−0.301	−1.67	0.106			
Sex (F-M)	0.008	0.030	0.136	0.25	0.801			

## Data Availability

The data presented in this study are available on request from the corresponding author. The data are not publicly available due ethical approval restrictions.

## Appendix A

**Table A1:** Overview of the direction and timing of kinematic changes between loading conditions.

Joint Angle	0%BM–20%BM		0%BM–40%BM		20%BM–40%BM	
	%Cycle	Direction	%Cycle	Direction	%Cycle	Direction
**Hip flexion-extension**	8–18	↑	20–39	↑	-	-
	58–59	↓	-	-	-	-
	88–100	↑	-	-	-	-
**Knee flexion-extension**	46–61	↓	8–11	↑	48–63	↓
	70–80	↑	46–64	↓	-	-
	95–98	↑	92–100	↑	-	-
**Ankle plantar-dorsi flexion**	49–62	↑	50–63	↑	-	-

↑: Increase in magnitude relative to lower mass; ↓: Decrease in magnitude relative to lower mass.

**Table A2:** Overview of the direction and timing of kinetic changes between loading conditions.

Joint Moment	0%BM–20%BM		0%BM–40%BM		20%BM–40%BM	
	%Stance	Direction	%Stance	Direction	%Stance	Direction
**Hip flexion-extension**			61–95	↑ flex	71–83	↑ flex
**Knee flexion-extension**	12–40	↑ ext	8–47	↑ ext	10–44	↑ ext
			88–97	↑ ext	91–96	↑ ext
**Ankle plantar-dorsi flexion**	48–95	↑ PF	43–95	↑ PF	57–86	↑ PF
**Vertical ground reaction force**	4–100%	↑	1–100%	↑	2–97%	↑

↑: Increase in magnitude relative to lower mass; flex: flexion; ext: extension; PF: plantarflexion.
